# Analysis of Drainage Volume in External Ventricular Drainage Based on Intracranial Pressure and Drainage Catheter Size for Clinical Nurses

**DOI:** 10.3390/healthcare13091009

**Published:** 2025-04-27

**Authors:** Hanna Lee, Boeun Yang, Kyeongeun Lee, Jeongwon Han

**Affiliations:** 1 Department of Nursing, Gangneung-Wonju National University, Wonju 25457, Republic of Korea; hannalee@gwnu.ac.kr; 2 College of Nursing Science, Kyung Hee University, Seoul 02447, Republic of Korea; 90471361@khu.ac.kr (B.Y.); keun7772@khu.ac.kr (K.L.)

**Keywords:** external ventricular drainage, flow rate, intracranial pressure, ventricular drainage

## Abstract

**Introduction:** The purpose of this study is to provide foundational data for nursing care in patients with external ventricular drainage (EVD) by comparatively analyzing drainage volume in relation to intracranial pressure (ICP) and drainage catheter size. **Methods:** In this study, we conducted a volumetric analysis using the continuity and Bernoulli equations, considering friction forces under predefined conditions. In adults in the supine position with 37 °C CSF, the ventricular drainage volume was assessed based on the height of the EVD system, ICP levels, and EVD catheter sizes. **Results:** The results indicated that the CSF flow rate increased with larger catheter diameters and when the EVD system was positioned lower than the reference point (foramen of Monro). Across all catheter sizes, the minimum CSF flow occurred when the EVD system was 15 cm above the reference point, while the maximum flow was observed when it was 15 cm below the reference point. This multidisciplinary study, utilizing fluid dynamics, quantitatively estimates the drainage volume in EVD systems based on ICP and catheter size, contributing to the nursing care of EVD systems. The findings underscore the importance of developing specific nursing guidelines to improve patient safety in external ventricular drainage management and incorporating them into clinical education. **Conclusions:** A limitation of this study is that it does not compare with patients in clinical settings for clinical empirical validity. Therefore, a stepwise validation process is necessary. So, future studies will need to compare medical record data with the results of this study to confirm the validity of the equations presented.

## 1. Introduction

External ventricular drainage (EVD) is a common neurosurgical procedure that involves inserting a catheter into the brain’s ventricles and connecting it to an external drainage system via a three-way connector. This setup facilitates cerebrospinal fluid (CSF) drainage and allows monitoring of intracranial pressure (ICP) [1]. EVD is typically employed in cases such as subarachnoid hemorrhage (SAH), intracranial hemorrhage (ICH), intraventricular hemorrhage (IVH), and hydrocephalus (HCP) caused by traumatic brain injury (TBI) [2,3]. There are two primary methods of EVD: continuous and intermittent drainage1. Continuous drainage keeps the catheter open to maintain stable ICP but carries a higher risk of infection and potential ventricular collapse due to excessive CSF drainage [4,5]. Conversely, intermittent drainage involves opening the catheter based on patient conditions, reducing the risks of infection and collapse but potentially allowing for ICP elevation when drainage is paused [6,7]. Healthcare providers must carefully consider the advantages and disadvantages of each method to determine the optimal drainage strategy based on the patient’s condition [1,8].

The EVD nursing guidelines for safe CSF drainage and management emphasize that nurses should verify the foramen of Monro (interventricular foramen) as the reference point when zeroing for ICP measurement, as recommended by the Korean Hospital Nurses Association [9]. Neurosurgeons are advised to establish the EVD fixation level and maintain the patient’s head in a neutral position with the bed elevated to 30 degrees. However, specific guidance regarding CSF drainage volume in relation to ICP and the EVD system height is not provided. Similarly, U.S. clinical guidelines focus on ensuring consistency in reference points (e.g., the foramen of Monro or the outer canthus of the eye) for ICP measurements and highlight the importance of nursing care during ICP monitoring and tube removal. However, they lack detailed recommendations regarding CSF drainage volume management [10]. Given the risks associated with EVD, such as hemorrhage, infection, decreased consciousness, cognitive impairments, and neurological sequelae, there is a pressing need for more specific and clear information on nursing care for patients with EVD [11,12]. Nurses often consult with physicians regarding the target CSF drainage volume and monitor the drainage status. This process can be challenging when there are significant variations in drainage volume or when drainage is not smooth [9,10,11]. Providing data that allow nurses to predict drainage rates based on key influencing factors could help them manage patients safely and perform their duties more effectively [10].

Among the factors influencing EVD drainage, ICP is a critical determinant. Normal ICP ranges from 5 to 15 mmHg, while levels above 20 mmHg are classified as increased ICP (IICP) [1,3,13]. Monitoring ICP is essential for identifying rapid cerebral edema progression10. CSF is a clear fluid typically maintained at 37 °C [14], and when ICP is within normal limits and CSF characteristics are stable, over 200 mL of CSF can be drained within 24 h. However, changes in the protein or blood concentration in CSF can affect drainage volume and flow rate [15]. For example, high protein concentrations increase CSF viscosity, reducing the flow rate by approximately 7%. Although this reduction has limited statistical or clinical significance, it must still be considered [16]. The drainage tube characteristics also affect the CSF flow rate. According to Poiseuille’s Law, which describes the flow of a viscous fluid through a cylindrical pipe, the flow rate depends on the pipe’s length, radius, fluid viscosity, and pressure difference [17]. While this law applies perfectly only under ideal conditions (e.g., constant viscosity, laminar flow, and uniform pipe diameter), it provides a useful approximation in many practical cases. Since CSF behaves as a Newtonian fluid, its drainage volume can vary depending on the selected catheter characteristics and ICP, even under the same patient conditions, necessitating appropriate nursing care.

Although neurosurgeons perform EVD surgery, nurses are primarily responsible for postoperative monitoring and maintenance of the EVD system [11]. Nurses need to assess a wide range of factors, including changes in ICP and neurological status, CSF drainage volume and characteristics, insertion site condition, CSF leakage, and any catheter kinking or obstruction [12]. This study aims to provide foundational data for EVD nursing care by comparatively analyzing drainage volume based on ICP and catheter size, offering insights to support effective management of EVD patients.

## 2. Materials and Methods

### 2.1. Methods

#### 2.1.1. Study Design

This study employs a control volume analysis to evaluate the drainage volume in EVD systems based on ICP and drainage tube size. The aim is to provide foundational data to support nursing care for patients with EVD.

#### 2.1.2. Subject of Interpretation

**Figure 1:**
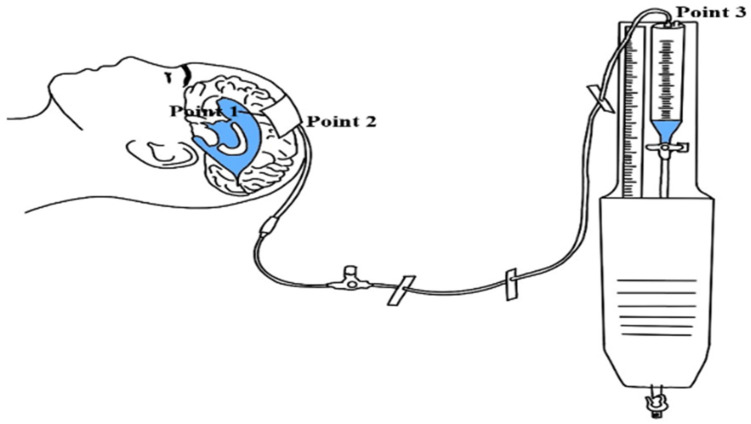
The structure of EVD. Point 1: the end point of the EVD catheter, Point 2: the EVD catheter connects to the circuit, Point 3: the circuit connects to the drip chamber.

The structural framework of this study is illustrated in Figure 1.

#### 2.1.3. Intracranial Pressure

ICP was set at the lower limit of the normal range (5 mmHg), the upper limit of the normal range (15 mmHg), and to represent IICP (20 mmHg) in a supine adult position [1,3].

#### 2.1.4. Cerebrospinal Fluid

The temperature of CSF was standardized at 37 °C, consistent with findings from previous studies [14]. CSF is composed primarily of water with small quantities of proteins, glucose, and electrolytes. Variations in protein, glucose, and red blood cell (RBC) concentrations within clinically observed ranges have been shown to exert minimal effects on the density and viscosity of CSF [18].

#### 2.1.5. External Ventricular Drainage Catheter

EVD catheters (Sewoon Medical Co. Ltd., Seoul, Republic of Korea) were utilized, including 6.0 Fr, 7.5 Fr, 9.0 Fr, 10.5 Fr, 12.0 Fr, and 14.0 Fr sizes.

#### 2.1.6. Height of the EVD System

The reference point for the EVD system was set at the foramen of Monro, designated as Point 1. The starting point of the EVD catheter was marked as Point 2. This reflects standard practice, where the catheter is inserted through a burr hole in the right frontal region of the skull and threaded subcutaneously, exiting the skin approximately 5 cm from the insertion to connect with the external drainage system [10,19]. Previous studies suggest perforating and inserting the EVD catheter 2–3 cm laterally from the height of the foramen of Monro [19]. Based on this, the vertical distance between the reference point (foramen of Monro) was set as h1. The start of the drip chamber, located parallel to the foramen of Monro, was designated as h2 with a value of 0 cm. In this study, the height of the EVD system (h3) refers to the vertical distance from the reference point (foramen of Monro) to the start of the drip chamber.

### 2.2. Data Collection

The study data were collected through a literature review, information provided by the manufacturer, and direct measurements. The specific parameters are described below.

#### 2.2.1. Intracranial Pressure

In this study, ICP was set to 666.612 Pa (5 mmHg) for the lower limit of the normal range, 1999.836 Pa (15 mmHg) for the upper limit of the normal range, and 2666.440 Pa (20 mmHg) to represent increased intracranial pressure (IICP) in a supine adult position [1,3].

#### 2.2.2. Density and Viscosity of Cerebrospinal Fluid

This study used a CSF at 37 °C density (ρ) of 1000.3 kg/m^3^ and viscosity (μ) of 0.0007 Pa/s, as established in prior research [18,20,21]. In this study, the viscosity of the CSF was set based on previous research finding [18], which indicated that there are no significant differences in CSF viscosity depending on its composition.

#### 2.2.3. Length and Inner Diameter of External Ventricular Drainage Catheter

The EVD catheter used in this study is manufactured by Sewoon Medical Co., Ltd. and certified as a nationally approved medical product (KS P ISO 20698) [22]. The EVD system includes the catheter, a connecting circuit, and a CSF collection bag. The internal diameter of the fluid-conducting tube is a key factor in flow analysis [23]. The catheter, a thin tube inserted directly into the ventricles, has internal diameters ($d_{1}$) of 6.0 Fr (1.0 mm), 7.5 Fr (1.5 mm), 9.0 Fr (1.7 mm), 10.5 Fr (2.1 mm), 12 Fr (2.5 mm), and 14 Fr (3.0 mm). All catheters are 360 mm in length ($L_{\alpha }$). The internal diameter of the circuit connecting the catheter to the drip chamber ($d_{2}$) is identical to that of the catheter itself, with a total circuit length of 1800 mm ${(L}_{\beta }$).

#### 2.2.4. Height of the EVD System

With reference to the vertical distance between the reference point (foramen of Monro) and the starting point of the EVD catheter was set to 14 cm ($h_{1}$) [24], as depicted in Figure 1. The start of the drip chamber, located parallel to the foramen of Monro with a value of 0 cm ($h_{2}$). In this study, the height of the EVD system refers to the vertical distance from the reference point (foramen of Monro) to the start of the drip chamber. Heights below the reference point were recorded as negative values, heights at the same level were set to 0 cm, and heights above the reference point were recorded as positive values, ranging from −15 to +15 cm ${(h}_{2}$). Previous research established the EVD system height by zeroing the horizontal level between the reference point and the start of the drip chamber, then adjusting heights from 0 to 15 cm [25]. However, since the height of the EVD system significantly impacts drainage volume, adjustments to the height may be necessary when CSF drainage decreases [10]. In this study, the final height of the EVD system was determined by establishing a height difference relative to the zero point.

### 2.3. Data Analysis

#### 2.3.1. Assumptions

In fluid dynamics, materials are broadly classified into solids and fluids [26]. Solids can resist shear force (force per unit area) to some extent, while fluids cannot resist even minor shear forces and continue to move and deform under shear stress [23]. The movement of fluids is termed fluid flow, which can be categorized into laminar and turbulent flow based on its characteristics [27]. Laminar flow refers to the orderly flow where fluid particles maintain consistent layers while moving at low speeds with low density and high viscosity. In contrast, turbulent flow involves irregular mixing and change when fluid with high density and low viscosity moves rapidly [28]. Laminar flow is dominated by viscous forces compared to inertial forces, while turbulent flow is dominated by inertial forces over viscous forces [23]. The distinction between laminar and turbulent flow depends on fluid velocity, viscosity, density, and the diameter of the tube through which the fluid flows. It is determined by the Reynolds number, a dimensionless function (Equation (1)). A Reynolds number below 2000 indicates laminar flow, a range of 2000–4000 indicates a transition between laminar and turbulent flow, and a number exceeding 4000 indicates turbulent flow [26].

(1) $$Re=\frac{\rho VD}{\mu }=\frac{VD}{\nu }$$

Here, ρ represents the fluid density, V the average fluid velocity, μ the kinematic viscosity, ν the dynamic viscosity, and D the diameter of the tube. The Bernoulli equation is an energy equation that indicates the conservation of total energy as fluid flows. In other words, it suggests that the forces of pressure, kinetic energy, and potential energy remain constant during fluid flow, as expressed in Equation (2).

(2) $$p+\frac{\rho V^{2}}{2}+\rho gz$$

where p denotes pressure, g represents gravitational acceleration, which is taken as 9.81 m/s^2^ in the expansion of the equation, and z is height. The Bernoulli equation holds under specific conditions, requiring steady state, incompressibility, frictionless flow, and applicability to a single streamline, with the assumption of no work or external heat transfer. Applying the standard form of the Bernoulli equation (Equation (2)) can lead to errors in analysis. To address these limitations in control volume analysis, a modified Bernoulli’s equation has been proposed, as shown in Equation (3).

(3) $$\frac{p_{1}}{\rho g}+\frac{\alpha {V_{1}}^{2}}{2g}+z_{1}=\frac{p_{2}}{\rho g}+\frac{\alpha {V_{2}}^{2}}{2g}+z_{2}+h_{f}$$

The modified Bernoulli equation accounts for energy loss due to frictional forces occurring during fluid movement. The kinetic energy correction factor (α) is 2.0 for laminar flow and 1.05 for turbulent flow. $h_{f}$ represents the energy lost due to friction as the fluid passes through the interior of a pipe. The head loss can be calculated using the Darcy-Weisbach equation (Equation (4)), where the Darcy friction factor (f) is determined by different equations for laminar (Equation (5)) and turbulent flow (Equation (6)).

(4) $$h_{f}=f\frac{L}{D}\frac{V^{2}}{2g}$$

(5) $$f=\frac{64}{Re}$$

(6) $$\frac{1}{f^{1/2}}=-2.0\;\log(\frac{\varepsilon /D}{3.7}+\frac{2.51}{Re\;f^{1/2}})$$

Here, L represents the length of the pipe, and ε denotes the pipe’s roughness. For the Bernoulli equation with friction to be valid in this study, the analysis must assume incompressible flow, steady state, and no energy transfer with the surroundings. In this study, CSF was used as the fluid at 37 °C. For a liquid to be compressible, it must have a speed close to the speed of sound (approximately 1500 m/s), however, the velocity of CSF entering the collection bag through the EVD catheter from the ventricles is within a range where compressibility can be neglected, which satisfies the incompressibility assumption. This study focuses on the continuous flow segment of CSF, excluding the initial unsteady state during catheter insertion into the ventricles, which fulfils the steady state assumption. Lastly, the assumption of no energy transfer with the surroundings is valid, as energy transfer between the EVD system and the external environment is minimal. Therefore, the assumptions for using the Bernoulli equation with friction considerations are suitable for the data analysis and interpretation in this study.

#### 2.3.2. Continuity Equation

Based on the above assumptions, this study employs the continuity equation and Bernoulli equation with friction considerations for control volume analysis. The continuity equation relevant to the subject of this study is expressed in Equation (7).

(7) $$A_{\alpha }V_{\alpha }=A_{\beta }V_{\beta }$$

(8) $${D_{\alpha }}^{2}V_{\alpha }={D_{\beta }}^{2}V_{\beta }$$

Here, the subscript α denotes the starting point of the EVD catheter, and the subscript β refers to the endpoint of the catheter (EVD circuit) connecting the EVD catheter and drip chamber. In Equation (7), the area of the circular cross-section of the conduit is expressed in terms of the square of the diameter, as shown in Equation (8).

#### 2.3.3. Friction-Adjusted Bernoulli’s Equation

The Bernoulli equation with friction considerations applicable to the subject of this study is as follows.

(9) $$\frac{P_{1}}{\rho g}+\frac{\alpha {V_{1}}^{2}}{2g}+h_{1}=\frac{P_{2}}{\rho g}+\frac{\alpha {V_{2}}^{2}}{2g}+h_{2}+h_{f\alpha }$$

(10) $$\frac{P_{2}}{\rho g}+\frac{\alpha {V_{2}}^{2}}{2g}+h_{2}=\frac{P_{3}}{\rho g}+\frac{\alpha {V_{3}}^{2}}{2g}+h_{3}+h_{f\beta }$$

Equations (9) and (10) are the Bernoulli equations with friction considerations applied to the EVD catheter and circuit, respectively. Here, subscript 1 denotes the starting point of the EVD catheter, subscript 2 indicates the starting point of the circuit, and subscript 3 signifies the endpoint of the circuit. In the α system, between points 1 and 2, $V_{1}=V_{2}$, and in the β system, between points 2 and 3, $V_{2}=V_{3}$ holds. $P_{3}$ is the pressure at the endpoint of the circuit and becomes zero. Therefore, Equations (9) and (10) can be simplified to Equations (11) and (12) as follows.

(11) $$\frac{P_{1}}{\rho g}+h_{1}=\frac{P_{2}}{\rho g}+h_{2}+h_{f\alpha }$$

(12) $$\frac{P_{2}}{\rho g}+h_{2}=h_{3}+h_{f\beta }$$

#### 2.3.4. Characteristic Equation

This study derived the characteristic equation by combining the continuity and Bernoulli equations with friction considerations to investigate the characteristics of ICP changes with posture and CSF flow rate concerning the size of the drainage tube. The characteristic equation represents the relationship among ICP, temperature of CSF, height of the EVD system, size of the EVD catheter, and flow rate of the drained CSF. Since Equations (11) and (12) must be satisfied simultaneously, they can be combined and expressed as Equation (13).

(13) $$\frac{P_{1}}{\rho g}+h_{1}=h_{3}+h_{f\alpha }+h_{f\beta }$$

By using the definition of the Darcy friction factor for laminar flow ($f=\frac{64}{Re}$), Equation (13) can be expressed as

(14) $$\frac{P_{1}}{\rho g}+\left(h_{1}-h_{2}\right)=\frac{64\mu }{\rho V_{\alpha }D_{\alpha }}\frac{L_{\alpha }}{D_{\alpha }}\frac{{V_{\alpha }}^{2}}{2g}+\frac{64\mu }{\rho V_{\beta }D_{\beta }}\frac{L_{\beta }}{D_{\beta }}\frac{{V_{\beta }}^{2}}{2g}\;=\frac{32\mu L_{\alpha }V_{\alpha }}{\rho D_{\alpha }g}+\frac{32\mu L_{\beta }V_{\beta }}{\rho {D_{\beta }}^{2}g}$$

From the continuity equation in Equation (8), $V_{\alpha }$ can be expressed as a function of $V_{\beta }$ as

(15) $$V_{\alpha }=\frac{{D_{\beta }}^{2}}{{D_{\alpha }}^{2}}V_{\beta }$$

By substituting Equation (15) into Equation (14), $V_{\alpha }$ from the continuity equation can be expressed as a function of $V_{\beta }$:

(16) $$\frac{P_{1}}{\rho g}+\left(h_{1}-h_{2}\right)=kV_{\beta }$$

Here, k is a constant and can be expressed as

(17) $$k=\frac{32\mu }{\rho g}(\frac{{D_{\beta }}^{2}L_{\alpha }}{{D_{\alpha }}^{4}}+\frac{L_{\beta }}{{D_{\beta }}^{2}})$$

#### 2.3.5. Reynolds Number

In this study, to verify the validity of the laminar flow assumption, the Reynolds number, as defined in Equation (1), was calculated using the velocities derived in Equations (16) and (17) to confirm that the flow is laminar.

## 3. Results

The analysis of drainage volume according to the height of the EVD system, accounting for ICP and catheter size in a supine adult with cerebrospinal fluid (CSF) at 37 °C, showed that the CSF flow rate increased with larger internal diameters of the catheter and lower system heights. The minimum flow rate for all catheter sizes was observed when the EVD system was 15 cm above the reference point (foramen of Monro), and the maximum flow rate occurred at 15 cm below the reference point. The results also demonstrated that for the same catheter size and system height, an increase in ICP resulted in a proportional increase in CSF drainage volume.

### 3.1. Ventricular Drainage Volume Based on the Height of the EVD System Considering ICP and EVD Catheter Size

**Figure 2:**
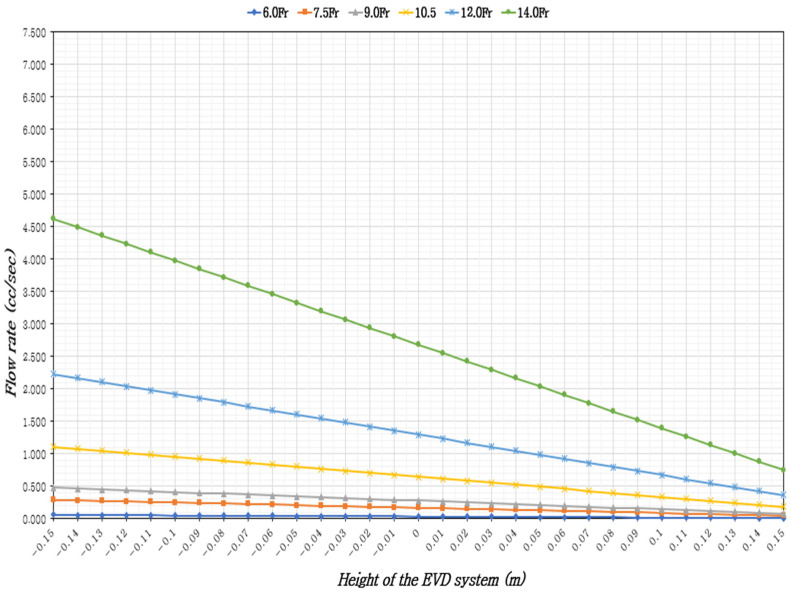
External ventricular drainage volume according to the height of the EVD system and the size of the EVD catheter, considering the minimum ICP (5 mmHg).

**Figure 3:**
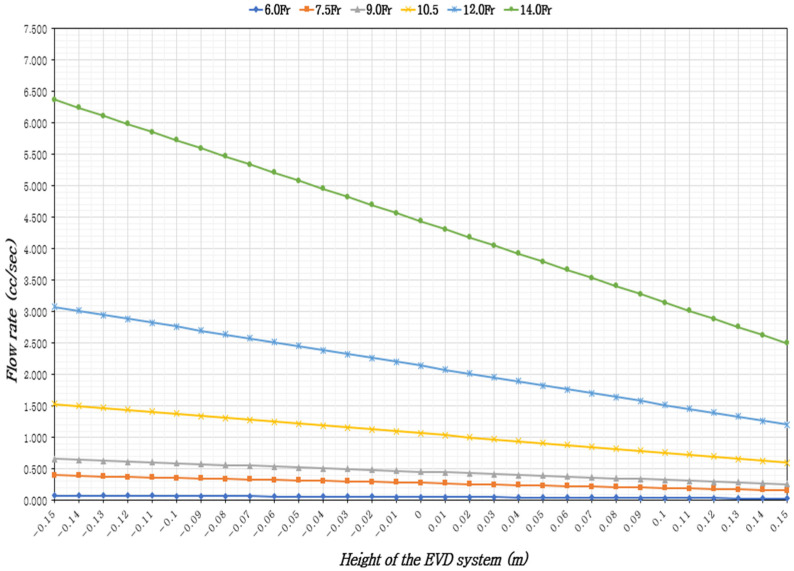
External ventricular drainage volume according to the height of the EVD system and the size of the EVD catheter, considering the maximum ICP (15 mmHg).

**Figure 4:**
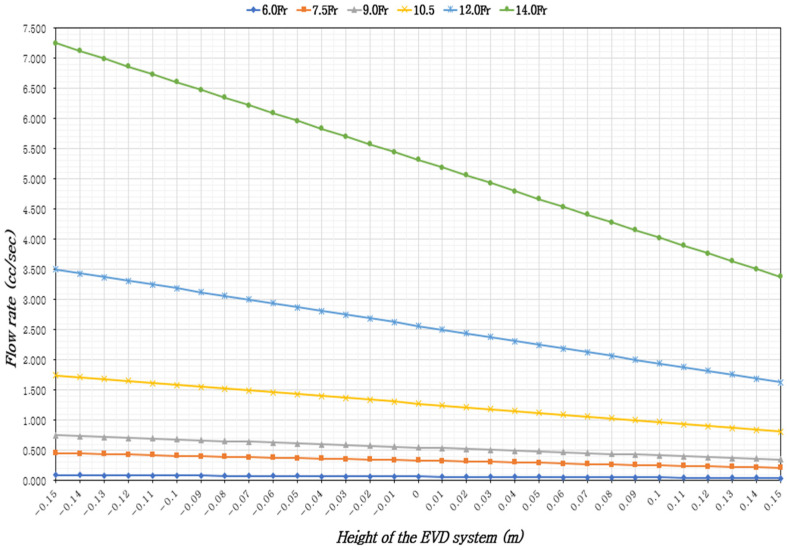
External ventricular drainage volume according to the height of the EVD system and the size of the EVD catheter, considering IICP (20 mmHg).

In adults in the supine position with 37 °C CSF, the ventricular drainage volume was assessed based on the height of the EVD system, ICP levels, and EVD catheter sizes. The results indicated that the CSF flow rate increased with larger catheter diameters and when the EVD system was positioned lower than the reference point (foramen of Monro). Across all catheter sizes, the minimum CSF flow occurred when the EVD system was 15 cm above the reference point, while the maximum flow was observed when it was 15 cm below the reference point (Table 1, Table 2 and Table 3), (Figure 2, Figure 3 and Figure 4).

At a minimum normal ICP of 5 mmHg, for a constant EVD catheter size, the CSF flow rate increased by 6.17 to 6.33 times when the EVD system was 15 cm below the reference point compared to 15 cm above. With a constant EVD system height, the flow rate increased by 81 to 83.1 times with larger catheter diameters. With the EVD system height fixed at 0 cm, the time to drain 5 cc of CSF varied by catheter size: approximately 2 m and 32 s for 6.0 Fr, 30 s for 7.0 Fr, 18 s for 9.0 Fr, 8 s for 10.5 Fr, 4 s for 12 Fr, and within 2 s for 14 Fr (Table 1), (Figure 2).

At a maximum normal ICP of 15 mmHg, with a constant catheter size, the CSF flow rate increased by up to 2.55 times when the EVD system was 15 cm below the reference point compared to 15 cm above. With a constant EVD system height, the CSF flow rate increased by 80.63 to 80.65 times with larger catheter diameters. With the EVD system height fixed at 0 cm, the time to drain 5 cc of CSF was approximately 1 min and 31 s for 6.0 Fr, 18 s for 7.0 Fr, 11 s for 9.0 Fr, 5 s for 10.5 Fr, 2 s for 12 Fr, and within 1 s for 14 Fr (Table 2), (Figure 3).

At an elevated ICP (IICP) of 20 mmHg, with a constant catheter size, the CSF flow rate increased by 2.12 to 2.15 times when the EVD system was 15 cm below the reference point compared to 15 cm above. With a constant EVD system height, the CSF flow rate increased by 80.4 to 81.43 times with larger catheter diameters. With the EVD system height fixed at 0 cm, the time to drain 5 cc of CSF was approximately 1 min and 16 s for 6.0 Fr, 15 s for 7.0 Fr, 9 s for 9.0 Fr, 4 s for 10.5 Fr, 2 s for 12 Fr, and within 1 s for 14 Fr (Table 3), (Figure 4).

### 3.2. Validity of Laminar Flow Assumption for CSF Flow

The validity of the laminar flow assumption for CSF flow was examined under 37 °C CSF conditions in adults in the supine position, with normal ICP of 5 mmHg and 15 mmHg, and elevated ICP (IICP) of 20 mmHg. This was assessed based on EVD catheter sizes and the height of the EVD system. The Reynolds number Reα from the EVD catheter start to the EVD circuit start (analysis points 1 to 2) confirmed laminar flow in most cases, except for specific conditions.

At an ICP of 5 mmHg, with a 14 Fr catheter and the EVD system positioned 5 to 15 cm below the reference point, the Reynolds number ${Re}_{\alpha }$ ranged from 2018 to 2800, indicating a transition zone. At an ICP of 15 mmHg, with a 12 Fr catheter and the EVD system positioned 10 to 15 cm below the reference point, the Reynolds number (${Re}_{\alpha }$) ranged from 2009 to 2236. In the same scenario, with a 14 Fr catheter and the system 15 cm below to 8 cm above the reference point, the Reynolds number ${Re}_{\alpha }$ ranged from 2064 to 3863, indicating a transition zone.

At an IICP of 20 mmHg, with a 12 Fr catheter and the system positioned 3 to 15 cm below the reference point, the Reynolds number ${Re}_{\alpha }$ ranged from 2000 to 2543. With a 14 Fr catheter across all EVD system heights, (${Re}_{\alpha }$) ranged from 2048 to 4395, indicating a transition zone, marking the shift from laminar to turbulent flow. Additionally, the Reynolds number (${Re}_{\beta }$) from the start to the end of the EVD circuit (analysis points 2 to 3) showed similar results to ${Re}_{\alpha }$.

To provide a clinically practical reference without the need for complex fluid dynamics equations, we present a summary table outlining the estimated time required to drain 5cc of cerebrospinal fluid (CSF) through EVD catheters of varying sizes, under standardized conditions: 37 °C CSF temperature, supine adult position, and EVD system height fixed at 0 cm relative to the Monro’s foramen (Table 4). This table is intended to support clinical decision-making by nurses and practitioners in adjusting EVD settings according to patient status and equipment characteristics. The data clearly demonstrate that CSF drainage becomes faster with larger catheter diameters and higher ICP levels. For instance, under an ICP of 15 mmHg, a 9.0 Fr catheter drains 5cc of CSF in approximately 18 s, compared to 1 min and 52 s with a 6.0 Fr catheter—a clinically significant difference.

## 4. Discussion

This study uses a control volume analysis to evaluate ventricular drainage volumes based on ICP and catheter size, highlighting several key findings.

First, the CSF drainage volume increases as the EVD system height decreases. When the EVD catheter size is constant and ICP is fixed, the CSF drainage volume increases when the EVD system is below the reference point (foramen of Monro). This supports EVD nursing guidelines [9,10], which recommend adjusting the EVD system position based on patient posture and condition changes. Even a 1 cm decrease in EVD system height can increase CSF drainage by 0.1 to 7.74 cc per minute under IICP conditions, depending on catheter size. These findings emphasize the need for precise height adjustments to manage ICP and CSF drainage accurately, which is crucial for patient stability and reducing complication risks. Therefore, height adjustments should be made whenever the patient’s position changes, requiring clear communication among healthcare staff and continuous monitoring. Adjusting the EVD height during continuous drainage should be done cautiously and tailored to the patient’s condition. This study offers clinically useful insights for estimating drainage volume based on height adjustments.

Second, the CSF flow rate increases with larger EVD catheter diameters. With a constant EVD system height, the CSF flow rate increases by 81 to 83.1 times with larger catheter diameters, consistent with Poiseuille’s law, which states that laminar flow velocity is proportional to the fourth power of the tube radius [17]. Previous studies also show that flow increases with larger diameters [29]. When the EVD system height is fixed at 0 cm, the time to drain 5 cc of CSF varies significantly by catheter size: approximately 2 min and 32 s for 6.0 Fr, 30 s for 7.0 Fr, 18 s for 9.0 Fr, 8 s for 10.5 Fr, 4 s for 12 Fr, and within 2 s for 14 Fr. Therefore, managing flow through detailed adjustments to the EVD system is essential, especially for larger diameter catheters, where automated pressure-flow control systems can minimize flow variability. Strict flow control protocols should be followed when using large-diameter catheters.

Third, the study indicates that, for constant EVD catheter size and system height, increasing ICP leads to greater CSF drainage. This aligns with the principle that higher pressure in the drainage tube increases flow. Continuous ICP monitoring is vital for conditions like SAH, ICH, TBI, HCP, and brain tumors to predict CSF drainage volumes [30,31,32]. However, the flow change due to increased ICP is relatively small compared to the change from catheter diameter. For instance, under IICP conditions, increasing the catheter diameter from 6.0 Fr to 14.0 Fr resulted in an increase in CSF drainage from 80.97 times to 81.05 times. Nurses managing EVD patients should be aware of both ICP and catheter size to predict drainage volumes accurately. Previous studies [33,34] report challenges when CSF drainage occurs more rapidly than expected or when drainage is not smooth, impacting workload and patient safety. Clear guidelines on these issues could enhance nursing care efficiency. Additionally, developing alert systems or equipment to notify healthcare professionals when preset drainage targets are met would be beneficial. Especially in IICP patients, effective CSF drainage management is crucial for patient outcomes, potentially reducing nurse workload and increasing patient safety.

This interdisciplinary study contributes to EVD system nursing by estimating ventricular drainage volumes based on ICP and catheter size. Drainage volume control plays a key role in ICP management and patient stabilization, underscoring the need for data-driven approaches. A limitation of this study is that it does not compare with actual patients in clinical settings for clinical empirical validity. Patients with EVD applications are considered high-risk subjects even among study populations. Therefore, a stepwise validation process is necessary. So, future studies will need to compare medical record data with the results of this study to confirm the validity of the equations presented. Once validity is established, the equations will be applied to actual patients to further provide foundational data for clinical nurses managing EVD. Additionally, considering that CSF can contain various substances such as blood or proteins, it is necessary to develop a Computational Fluid Dynamics (CFD) model based on the data from this study for more accurate simulation research. Although this study only considered continuous drainage situations, a CFD analysis could also verify the differences between intermittent and continuous drainage. Moreover, future research should focus on validating the fluid dynamic models and drainage estimation methods in clinical settings, developing automated and smart EVD management technologies (e.g., real-time flow control and ICP monitoring systems to prevent over drainage when using larger catheter sizes), and designing specific nursing guidelines for safer EVD management.

## 5. Conclusions

This study analyzes ventricular drainage volumes based on ICP and catheter size to provide foundational data for nursing care of EVD patients. The results show that CSF flow increases with higher ICP, larger catheter diameters, and lower EVD system heights relative to the reference point (foramen of Monro) in adults with 37 °C CSF. Further analysis of CSF flow in varying ICP conditions and additional research on transition zones are needed to refine EVD nursing practices. In existing nursing research and practice guidelines, the management of EVD has primarily focused on the height of the drainage system. However, considering the variations in drainage velocity and volume observed by clinical nurses, it is necessary to investigate factors from various perspectives in addition to those presented in this study. Especially this study was conducted as a preliminary study before confirming clinical validity. Therefore, based on the results of this study, future studies are needed in subjects with homogeneous neurological characteristics or in situations by disease (e.g., intracranial tumor, postoperative ventriculostomy), and considering ICP monitors, Ommaya reservoirs, etc.

## Figures and Tables

**Table 1:** The EVD volume according to the height of the EVD system and the size of the EVD catheter at 5 mmHg ICP (flow unit: cc/s).

The Height of the EVD System (m)	CSF Temperature 37 °C					
	The Size of the EVD Catheter (Fr)					
	6.0	7.5	9.0	10.5	12.0	14.0
−0.15	0.057	0.289	0.476	1.109	2.227	4.617
−0.14	0.055	0.281	0.463	1.078	2.164	4.488
−0.13	0.054	0.272	0.449	1.047	2.102	4.359
−0.12	0.052	0.264	0.436	1.016	2.040	4.230
−0.11	0.051	0.256	0.423	0.985	1.978	4.101
−0.10	0.049	0.248	0.410	0.954	1.916	3.972
−0.09	0.047	0.240	0.396	0.923	1.853	3.843
−0.08	0.046	0.232	0.383	0.892	1.791	3.714
−0.07	0.044	0.224	0.370	0.861	1.729	3.585
−0.06	0.043	0.216	0.356	0.830	1.667	3.456
−0.05	0.041	0.208	0.343	0.799	1.605	3.327
−0.04	0.039	0.200	0.330	0.768	1.542	3.198
−0.03	0.038	0.192	0.316	0.737	1.480	3.069
−0.02	0.036	0.184	0.303	0.706	1.418	2.940
−0.01	0.035	0.176	0.290	0.675	1.356	2.811
0	0.033	0.168	0.277	0.644	1.294	2.682
0.01	0.032	0.160	0.263	0.613	1.231	2.553
0.02	0.030	0.152	0.250	0.582	1.169	2.424
0.03	0.028	0.143	0.237	0.551	1.107	2.295
0.04	0.027	0.135	0.223	0.520	1.045	2.166
0.05	0.025	0.127	0.210	0.489	0.983	2.037
0.06	0.024	0.119	0.197	0.458	0.920	1.908
0.07	0.022	0.111	0.183	0.427	0.858	1.779
0.08	0.020	0.103	0.170	0.396	0.796	1.650
0.09	0.019	0.095	0.157	0.365	0.734	1.521
0.10	0.017	0.087	0.144	0.334	0.672	1.392
0.11	0.016	0.079	0.130	0.303	0.609	1.263
0.12	0.014	0.071	0.117	0.272	0.547	1.134
0.13	0.012	0.063	0.104	0.241	0.485	1.005
0.14	0.011	0.055	0.090	0.210	0.423	0.876
0.15	0.009	0.047	0.077	0.179	0.360	0.748

**Table 2:** The EVD volume according to the height of the EVD system and the size of the EVD catheter at 15 mmHg ICP (flow unit: cc/s).

The Height of the EVD System (m)	CSF Temperature 37 °C					
	The Size of the EVD Catheter (Fr)					
	6.0	7.5	9.0	10.5	12.0	14.0
−0.15	0.079	0.398	0.657	1.529	3.072	6.370
−0.14	0.077	0.390	0.644	1.498	3.010	6.241
−0.13	0.075	0.382	0.630	1.468	2.948	6.112
−0.12	0.074	0.374	0.617	1.437	2.885	5.983
−0.11	0.072	0.366	0.604	1.406	2.823	5.854
−0.10	0.071	0.358	0.590	1.375	2.761	5.725
−0.09	0.069	0.350	0.577	1.344	2.699	5.596
−0.08	0.067	0.342	0.564	1.313	2.637	5.467
−0.07	0.066	0.334	0.550	1.282	2.574	5.338
−0.06	0.064	0.326	0.537	1.251	2.512	5.209
−0.05	0.063	0.318	0.524	1.220	2.450	5.080
−0.04	0.061	0.309	0.511	1.189	2.388	4.951
−0.03	0.060	0.301	0.497	1.158	2.326	4.822
−0.02	0.058	0.293	0.484	1.127	2.263	4.693
−0.01	0.056	0.285	0.471	1.096	2.201	4.564
0	0.055	0.277	0.457	1.065	2.139	4.435
0.01	0.053	0.269	0.444	1.034	2.077	4.306
0.02	0.052	0.261	0.431	1.003	2.015	4.177
0.03	0.050	0.253	0.417	0.972	1.952	4.048
0.04	0.048	0.245	0.404	0.941	1.890	3.919
0.05	0.047	0.237	0.391	0.910	1.828	3.790
0.06	0.045	0.229	0.378	0.879	1.766	3.661
0.07	0.044	0.221	0.364	0.848	1.703	3.532
0.08	0.042	0.213	0.351	0.817	1.641	3.403
0.09	0.040	0.205	0.338	0.786	1.579	3.274
0.10	0.039	0.197	0.324	0.755	1.517	3.145
0.11	0.037	0.189	0.311	0.724	1.455	3.016
0.12	0.036	0.180	0.298	0.693	1.392	2.887
0.13	0.034	0.172	0.284	0.662	1.330	2.758
0.14	0.032	0.164	0.271	0.631	1.268	2.629
0.15	0.031	0.156	0.258	0.600	1.206	2.500

**Table 3:** The EVD volume according to the height of the EVD system and the size of the EVD catheter at 20 mmHg ICP (flow unit: cc/s).

The Height of the EVD System (m)	CSF Temperature 37 °C					
	The Size of the EVD Catheter (Fr)					
	6.0	7.5	9.0	10.5	12.0	14.0
−0.15	0.089	0.453	0.747	1.740	3.495	7.247
−0.14	0.088	0.445	0.734	1.709	3.432	7.118
−0.13	0.086	0.437	0.721	1.678	3.370	6.989
−0.12	0.085	0.429	0.707	1.647	3.308	6.860
−0.11	0.083	0.421	0.694	1.616	3.246	6.731
−0.10	0.082	0.413	0.681	1.585	3.184	6.602
−0.09	0.080	0.405	0.667	1.554	3.121	6.473
−0.08	0.078	0.396	0.654	1.523	3.059	6.344
−0.07	0.077	0.388	0.641	1.492	2.997	6.215
−0.06	0.075	0.380	0.628	1.461	2.935	6.086
−0.05	0.074	0.372	0.614	1.430	2.873	5.957
−0.04	0.072	0.364	0.601	1.399	2.810	5.828
−0.03	0.070	0.356	0.588	1.368	2.748	5.699
−0.02	0.069	0.348	0.574	1.337	2.686	5.570
−0.01	0.067	0.340	0.561	1.306	2.624	5.441
0	0.066	0.332	0.548	1.275	2.562	5.312
0.01	0.064	0.324	0.534	1.244	2.499	5.183
0.02	0.062	0.316	0.521	1.213	2.437	5.054
0.03	0.061	0.308	0.508	1.182	2.375	4.925
0.04	0.059	0.300	0.495	1.151	2.313	4.796
0.05	0.058	0.292	0.481	1.121	2.251	4.667
0.06	0.056	0.284	0.468	1.090	2.188	4.538
0.07	0.054	0.276	0.455	1.059	2.126	4.409
0.08	0.053	0.267	0.441	1.028	2.064	4.280
0.09	0.051	0.259	0.428	0.997	2.002	4.151
0.10	0.050	0.251	0.415	0.966	1.940	4.022
0.11	0.048	0.243	0.401	0.935	1.877	3.893
0.12	0.046	0.235	0.388	0.904	1.815	3.764
0.13	0.045	0.227	0.375	0.873	1.753	3.635
0.14	0.043	0.219	0.362	0.842	1.691	3.506
0.15	0.042	0.211	0.348	0.811	1.629	3.377

**Table 4:** Estimated CSF drainage time for 5cc at 0 cm EVD system height. (Conditions: 37 °C CSF, supine adult position).

	Cathter Size (Fr)	6 Fr	7.5 Fr	9 Fr	10.5 Fr	12 Fr	14 Fr
ICP							
5 mmHg		2′53″	50″	30″	13″	6″	3″
15 mmHg		1′52″	30″	18″	8″	4″	2″
20 mmHg		1′26″	25″	15″	7″	3″	2″

′: minute, ″: second.

## Data Availability

The data presented in this study are available on request from the corresponding author.

## Abbreviations

The following abbreviations are used in this manuscript:

CSF	Cerebrospinal fluid
EVD	External ventricular drainage
HCP	Hydrocephalus
IICP	Increased Intracranial pressure
ICH	Intracranial hemorrhage
ICP	Intracranial pressure
IVH	Intraventricular hemorrhage
SAH	Subarachnoid hemorrhage
TBI	Traumatic brain injury
